# Computational analysis of spliced leader trans-splicing in the regenerative flatworm Macrostomum lignano reveals its prevalence in conserved and stem cell related genes

**DOI:** 10.18699/VJ21.012

**Published:** 2021-02

**Authors:** K.V. Ustyantsev, E.V. Berezikov

**Affiliations:** Institute of Cytology and Genetics of Siberian Branch of the Russian Academy of Sciences, Novosibirsk, Russia; Institute of Cytology and Genetics of Siberian Branch of the Russian Academy of Sciences, Novosibirsk, Russia

**Keywords:** flatworms, regeneration, splicing, trans-splicing, neoblasts, spliced leader, Macrostomum lignano, плоские черви, регенерация, сплайсинг, транс-сплайсинг, необласты, сплайс-лидер, Macrostomum lignano.

## Abstract

In eukaryotes, trans-splicing is a process of nuclear pre-mRNA maturation where two different RNA molecules are joined together by the spliceosomal machinery utilizing mechanisms similar to cis-splicing. In diverse taxa of lower eukaryotes, spliced leader (SL) trans-splicing is the most frequent type of trans-splicing, when the same sequence derived from short small nuclear RNA molecules, called SL RNAs, is attached to the 5’ ends of different non-processed pre-mRNAs. One of the functions of SL trans-splicing is processing polycistronic pre-mRNA molecules transcribed from operons, when several genes are transcribed as one pre-mRNA molecule. However, only a fraction of trans-spliced genes reside in operons, suggesting that SL trans-splicing must also have some other, less understood functions. Regenerative flatworms are informative model organisms which hold the keys to understand the mechanism of stem cell regulation and specialization during regeneration and homeostasis. Their ability to regenerate is fueled by the division and differentiation of the adult somatic stem cell population called neoblasts. Macrostomum lignano is a flatworm model organism where substantial technological advances have been achieved in recent years, including the development of transgenesis. Although a large fraction of genes in M. lignano were estimated to be SL trans-spliced, SL trans-splicing was not studied in detail in M. lignano before. Here, we performed the first comprehensive study of SL trans-splicing in M. lignano. By reanalyzing the existing genome and transcriptome data of M. lignano, we estimate that 30 % of its genes are SL trans-spliced, 15 % are organized in operons, and almost 40 % are both SL trans-spliced and in operons. We annotated and characterized the sequence of SL RNA and characterized conserved cis- and SL transsplicing motifs. Finally, we found that a majority of SL trans-spliced genes are evolutionarily conserved and significantly over-represented in neoblast-specific genes. Our findings suggest an important role of SL trans-splicing in the regulation and maintenance of neoblasts in M. lignano.

## Abstract (ru)

Транс-сплайсинг у эукариот – это процесс созревания ядерных пре-мРНК, когда две различные молекулы РНК соединяются с помощью структур сплайсосомы по механизму, схожему с цис-сплайсингом. У различных таксонов низших эукариот наиболее распространенный тип транс-сплайсинга – сплайс-лидерный (СЛ) транс-сплайсинг, при котором одинаковая последовательность, происходящая от коротких малых ядерных РНК молекул, называемых СЛ РНК, присоединяется к 5’-концам различных непроцессированных пре-мРНК. Одна из функций СЛ транс-сплайсинга состоит в процессировании полицистронных молекул пре-мРНК, транскрибируемых с оперонов, когда транскрипция нескольких генов осуществляется как одна молекула пре-мРНК. Однако лишь часть генов, подвергающихся транс-сплайсингу, содержится в оперонах, что говорит о том, что у СЛ транссплайсинга должны быть и другие, менее изученные, функции. Регенерирующие плоские черви являются информативными модельными организмами, хранящими ключи к пониманию механизмов регуляции стволовых клеток и их дифференцировки во время регенерации и при гомеостазе. Их способность к регенерации – следствие деления и дифференцировки соматических стволовых клеток, называемых необластами, которые присутствуют у взрослых особей. Macrostomum lignano – модельный плоский червь, в исследованиях на котором в последние годы достигнут существенный технологический прогресс, включая разработку метода трансгенеза. Сплайс-лидерный транс-сплайсинг ранее не был детально изучен у M. lignano, хотя известно, что значительная часть генов M. lignano подвергается этому типу транс-сплайсинга. В настоящей работе мы осуществили первое обширное исследование СЛ транс-сплайсинга у M. lignano. Повторно проанализировав геномные и транскриптомные данные M. lignano, мы оцениваем, что 30 % его генов подвергаются СЛ транс-сплайсингу, 15 % расположены в оперонах, а почти 40 % находятся в оперонах и проходят через СЛ транс-сплайсинг. Мы провели аннотацию и охарактеризовали последовательность СЛ РНК и консервативных мотивов цис- и транс-сплайсинга. Обнаружено, что большинство генов, подвергающихся СЛ транс-сплайсингу, эволюционно консервативны и значительно перепредставлены в генах, специфичных для необластов. Наши результаты предполагают важную роль СЛ транс-сплайсинга в регуляции функционирования необластов у M. lignano

Before being used as templates for protein production, majority of RNA molecules transcribed in the nucleus (pre-mRNA) undergo three major modifications to become mature and fully functional mRNA. This is called RNA processing and it involves capping of the 5′ end, polyadenylation of the 3′ end, and splicing. Two types of splicing are distinguished – cis- and trans-splicing. During cis-splicing all the processing happens with the same pre-mRNA molecule, resulting in the removal of introns and merging of its exons. During trans-splicing, on the other hand, two different pre-mRNA molecules expressed from distinct genomic loci are joint into a new chimeric transspliced mRNA (Lasda, Blumenthal, 2011).

Trans-splicing was originally discovered in trypanosomes (Euglenozoa), where it was found that a short 39 bp leader sequence was post-transcriptionally attached to the 5′ ends of variant surface glycoproteins pre-mRNA (Boothroyd, Cross, 1982). Later, 5′ end addition of a 22 bp spliced leader (SL) was also observed in Caenorhabitis elegans mRNA of actin gene and some other genes (Krause, Hirsh, 1987). Now this process is well known as SL trans-splicing. A distinct feature of SL trans-splicing is that all such processed transcripts have the same short SL sequence, or its variant, at their 5′ ends. The SL sequence is derived from an exon of a non-coding small nuclear RNA molecule called SL RNA, which is ~100 nt in length and has 2,2,7-trimethylguanosine cap at its 5′ end instead of 7-methylguanosine cap, which is found in non-transspliced mRNAs (Liou, Blumenthal, 1990; Lasda, Blumenthal, 2011). SL RNAs have a splicing donor site at the exon 3′ end, while SL trans-spliced pre-mRNAs have a splicing acceptor site at the 5′ end of their first exon. SL trans-splicing results in removal of the 5′ non-exon pre-mRNA part called outron (Lasda, Blumenthal, 2011). It is experimentally shown that the only requirement for a gene to be predominantly SL trans-spliced is an acceptor splicing site close to the 5′ end of the first exon that is not complemented by a donor splicing site upstream in cis (Conrad et al., 1993). Thus comes another important feature of SL trans-splicing, namely that it allows formation and resolving of operons – adjacent genes transcribed as a single pre-mRNA from the same promoter region (Blumenthal, Gleason, 2003). However, apart from a clear function in polycistronic transcripts resolution, the function of SL trans-splicing for monocystronic transcripts is still in debate (Danks et al., 2015). It is hypothesized that the function may be in equalization of 5′ UTRs in length and their clearance from out-of-frame AUG start codons, while at the same time allowing less restricted evolution of 5′ upstream regulatory sequences, and in additional control of translation (Hastings, 2005; Danks, Thompson, 2015). So far, SL transsplicing was found in several clades of eukaryotes: dinoflagellates, euglenozoans, cnidarians, flatworms, nematodes, and ascidians (Lei et al., 2016). SL trans-splicing is most prominent in trypanosomes (100 % genes are trans-spliced) and in nematodes (70 % genes are trans-spliced) (Allen et al., 2011; Lei et al., 2016). 

Regenerative flatworms are informative models to understand the mechanism of stem cell regulation and specialization during regeneration and homeostasis. Their ability to regenerate is driven by the division and differentiation of the adult somatic stem cell population called neoblasts (Wagner et al., 2011; Mouton et al., 2018). Macrostomum lignano is the only flatworm species for which a method for stable transgenesis is available so far. The worm also has a number of features allowing for efficient cell lineage tracing and phenotype screening, which makes M. lignano an attractive model to study a wide range of biological processes (Grudniewska et al., 2016; Wudarski et al., 2017, 2019, 2020). Well-annotated M. lignano genome and transcriptome assemblies were recently published (Wudarski et al., 2017; Grudniewska et al., 2018). It was estimated that almost 21 % of its genes are SL trans-spliced to the same 35 bp SL sequence (Grudniewska et al., 2018). However, trans-splicing was not studied in details in M. lignano, and its impact on the genome functioning and maintenance is still unknown. Here, we present the first comprehensive study of SL trans-splicing in M. lignano and show that it is strongly connected with genes specific for the neoblasts of the worm.

## Materials and methods

**Data.** The published M. lignano genome Mlig_3_7 (Wudarski et al., 2017) and transcriptome Mlig_RNA_3_7_DV1_v3 (Grudniewska et al., 2018) assemblies and the corresponding annotation tracks were obtained from (http://gb.macgenome.org/downloads/Mlig_3_7/).

**Genome deduplication.** Mlig_3_7 genome assembly was deduplicated using purge_dups software (v1.0.1) with default settings (Guan et al., 2020) and utilizing published PacBio genome sequencing data (Wasik et al., 2015) for the calculation of contig coverages. Contig names from the deduplicated genome assembly were used to extract respective gene annotations from the full Mlig_3_7 genome annotation.

**Motif discovery and SL RNA annotation.** Presence of the SL sequence at the 5′ end of the M. lignano transcripts was established in the previous studies (Wasik et al., 2015; Grudniewska et al., 2016). For the annotation of trans-spliced genes, SL-containing RNA-seq reads were mapped to the Mlig_3_7 genome assembly and the presence of such reads at the beginning of transcripts was used as an evidence of SL trans-splicing (Wudarski et al., 2017; Grudniewska et al., 2018). Therefore, all the SL trans-spliced transcripts have the corresponding annotation in the Mlig_3_7 genome assembly, and the sequences upstream of their first exon were considered as outrons. Using the deduplicated annotation track of gene coordinates, we retrieved nucleotide sequences of genomic regions corresponding to exon-intron and exon-outron (for the trans-spliced genes) junctions with 50 bp flanks in both directions. All the sequences were converted to forward orientation and split into three groups corresponding to cis-donor, cis-acceptor, and trans-spliced acceptor sites. The sequences then were analysed for the presence of enriched motif using a stand-alone version of the DREME tool (Bailey, 2011). 

To determine the SL RNA gene sequence in the genome assembly, we used the 35 bp M. lignano SL sequence (CGG TCTCTTACTGCGAAGACTCAATTTA TTGCATG) as a seed for a BLASTn (Altschul et al., 1990) search requiring only 100 % matching hits. Next, we manually investigated genomic sequences surrounding the BLAST hits by matching the SL sequence track in the genome browser to the expected size of SL RNA (~100 bp). The corresponding sequences were then checked for folding into secondary structure canonical for SL RNA folding using Mfold web server (Zuker, 2003), and conserved motifs were then manually identified.

**Prediction of operons.** Intergenic distances were retrieved from the deduplicated genome annotation file. We only considered distances between immediately adjacent transcripts with the same transcriptional orientation and not interrupted by transcripts in opposite orientation. Distances were split into three categories: between SL trans-spliced genes, between a non-SL trans-spliced gene and an SL trans-spliced gene, and between non-SL trans-spliced genes. To adjust for repetitive element insertions, we retrieved the corresponding coordinates from the genome browser RepeatMasker and TRF tracks (http://gb.macgenome.org/) and subtracted them from the previously identified intergenic distances. Distribution of the distances was visualized as density plots using ggplot2 library in R.

After the analysis of the graphical data of the distances distributions, we selected the threshold value of 1000 bp, below which a pair of adjacent and SL trans-spliced genes were considered as belonging to the same operon. The same applies if the first gene is non-trans-spliced, but the second is SL transspliced. The distributions of lengths of operons of various sizes was visualized as violin plots using ggplot2 library in R.

**Estimation of gene conservation.** Gene annotation and data classifying genes as being specific to neoblasts or germline were retrieved from the previous study (Grudniewska et al., 2018). A gene was considered to be conserved if it has an open reading frame with a detectable homology to a human gene, which is indicated in its annotation, and non-conserved if lacking the homology to human, but has a predicted open reading frame with homology to proteins in other organisms. Otherwise, a gene was considered non-coding. 

## Results

**Deduplication of genome assembly.** The published Mlig_3_7 genome assembly is based on the sequencing data from DV1 M. lignano line. This line has a 2n = 10 karyotype (four large and six small chromosomes) and was demonstrated to have undergone a duplication of its large chromosome (Zadesenets et al., 2017), while the karyotype of the basal wild type population is 2n = 8 (two large and six small chromosomes) (Wudarski et al., 2017). The size of Mlig_3_7 assembly is 764 Mb, which corresponds to the experimental measurement of the genome size in the DV1 line (Wudarski et al., 2017), and the assembly contains the duplicated large chromosome sequences. To avoid gene overcounting due to the presence of these duplicated sequences in the Mlig_3_7 assembly, we removed the most redundant scaffolds by deduplicating Mlig_3_7 assembly using purge_dups software (Guan et al., 2020). This resulted in approximately 46 % drop in the number of scaffolds (from 5270 to 2841) and decreased the genome size to 580 Mb, which is close to the genome size measurements for the NL10 line of M. lignano, which does not have the chromosomal duplication (Wudarski et al., 2017). Next, we removed the records from transcriptome annotation which corresponded to the redundant scaffolds.

**Fig. 1.**
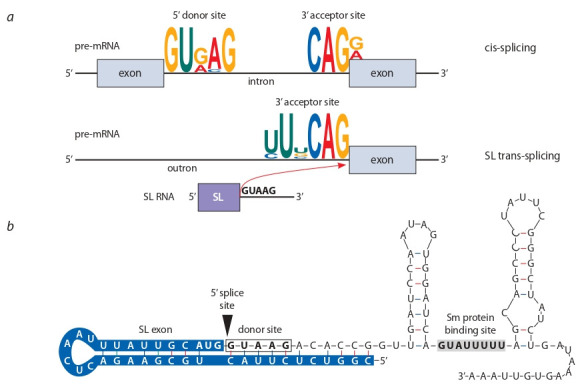
Features of cis-splicing and SL trans-splicing in M. lignano a – conserved motifs enriched at the splicing junction sites in cis-spliced and SL trans-spliced genes; b – sequence and predicted secondary structure of M. lignano SL RNA gene.

**Motif discovery and SL RNA gene mapping.** Investigation of the deduplicated part of the transcriptome shows that a significant fraction of genes, 21 754 out of 71 499 (30 %), are SL trans-spliced in M. lignano. This means that they all have the same 35 bp SL sequence (CGGTCTCTTACTGCG AAGACTCAATTTA TTGCATG) at the 5′ end of their processed transcripts (Wudarski et al., 2017; Grudniewska et al., 2018). Despite this, SL trans-splicing was not characterized in more detail in M. lignano. First, we retrieved genomic DNA sequences near the cis-splicing and SL trans-splicing exon-intron/exon-outron junction sites and checked if they are enriched for some motifs using DREME (Fig. 1, a) (Bailey, 2011). In total, we obtained 187 627 regions around 5′ donor and 3′ acceptor cis-splicing sites and 21 754 regions around SL trans-splicing sites. The first most enriched motifs near cis-splicing 5′ donor and 3′ acceptor sites were GT[G/A]AG (found in 122 399 regions, p-value: 8.8e–23 468) and CAG (found in 112 174 regions, p-value: 1.7e–12 459), respectively, corresponding to canonical cis-splicing motifs. A motif [T/C] TNCAG (found in 9551 regions, p-value: 1.3e–1631) was the top enriched motif near SL trans-splicing 3′ acceptor sites. All the motifs were positioned right at the exon-intron/exonoutron junctions of the corresponding sites (see Fig. 1, a)

Next, to confirm the presence of the SL RNA gene in the genome assembly, we analysed the secondary structure of the previously published sequence of M. lignano SL RNA from the ML2 version of the genome (Wasik et al., 2015). However, we found that the reported sequence was erroneously assigned as SL RNA, since it clearly maps to the 5′ end of an SL trans-spliced protein-coding gene (Mlig013257.g1, scaf577:45 663-48 770) in the Mlig_3_7 assembly, and also does not fold into canonical structure with three hairpin loops (data not shown) (Xie, Hirsh, 1998). Therefore, we decided to identify the actual SL RNA gene in the newer Mlig_3_7 assembly. Using SL sequence as a seed for the genomic BLASTn search, we mapped a 109 bp sequence, which is repeated eight times in the deduplicated genome and has the canonical SL RNA secondary structure predicted by Mfold web server (see Fig. 1, b) (Zuker, 2003). Subsequent sequence analysis showed clear signatures of an SL RNA: the SL sequence is at the 5′ end of the gene and forms the first hairpin loop, immediately after the SL sequence there is a clear 5′ donor splicing site (GTAAG), and between two other hairpin loops there is a motif similar to the binding site of Sm spliceosomal protein (see Fig. 1, b) (Ganot et al., 2004; Stover et al., 2006).

**Fig. 2.**
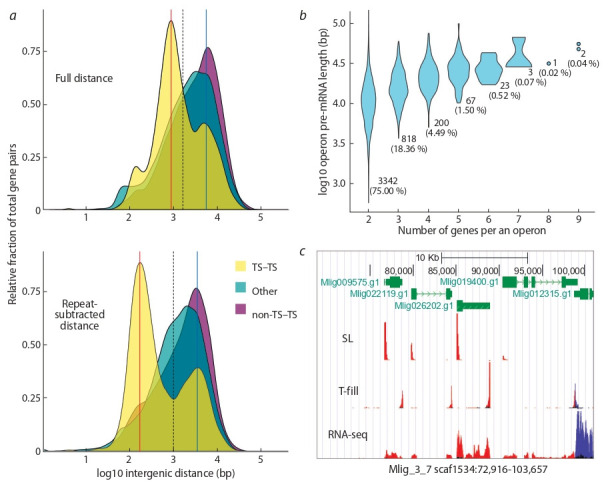
Identification and characteristics of genes in operons in M. lignano genome. a – distribution of intergenic distances between various gene types. TS – SL trans-spliced, non-TS – non-SL trans-spliced. Red and blue vertical lines indicate modes of the distributions. Vertical black dashed line indicates distance threshold value selected to separate genes in operons; b – putative pre-mRNA length and abundance of different operon sizes; c – an example of an operon with four genes as depicted in the M. lignano genome browser (http://gb.macgenome.org). Genes are in green, with exons as blocks and introns as dashed lines. Nonprotein-coding part of the exons are narrower. SL – RNA-seq reads mapped which contained the SL sequence at their 5’ ends (trimmed). T-fill – RNA-seq reads mapped containing mRNA 3’ poly-A ends. Reads mapped in forward orientation are in red, and the reversed reads are in blue.

**Operon analysis.** The important feature of SL transsplicing is that it allows for processing of long polycistronic pre-mRNA molecules expressed from a single promoter region in a way similar to prokaryote operons. In principle, genome-guided transcriptome assembly using RNA-seq data allows identification of such operons and their corresponding pre-mRNA sequences, which we previously annotated as transcriptional units (Wudarski et al., 2017; Grudniewska et al., 2018). However, it is not always possible to fully reconstruct an operon from RNA-seq data alone, since transcriptional units predicted from RNA-seq data tend to split in the repeat-rich intergenic regions of operons, where read coverage depends on both operon expression level and the frequency of repeats in the genome. Instead, to estimate what fraction of M. lignano genes are organized in operons based on their genomic organization, we first explored how intergenic distances between trans- and non-trans-spliced genes are distributed in M. lignano genome (Fig. 2, a). We found that distribution of distances between trans-spliced genes has multimodal distribution, while it is unimodal distribution for non-transspliced/trans-spliced and non-trans-spliced/non-trans-spliced intergenic distances (see Fig. 2, a). SL trans-splicing is an ancient evolutionary mechanism (Lei et al., 2016), which is mostly abundant in the genomes of simply organized organisms, which have low repetitive content and relatively small genomes (Gregory et al., 2007). We hypothesized that neutral accumulation of repeats could have influenced the distances between genes in the operons. Interestingly, after we adjusted the intergenic distances by subtracting the fraction occupied by repetitive sequences (simple repeats and transposable elements), it had the most impact on the distances between trans-spliced genes, revealing a clear bimodal distribution with the most prominent peak at around 100 bp (see Fig. 2, a). This observation indicates that repeats have a substantial contribution to intergenic distances in operons. To classify genes as belonging to the same operon, we decided to use the repeat-adjusted distances with a threshold value of 1 Kb, which separates the two modes of the intergenic distances between trans-spliced genes (see Fig. 2, a).

**Fig. 3.**
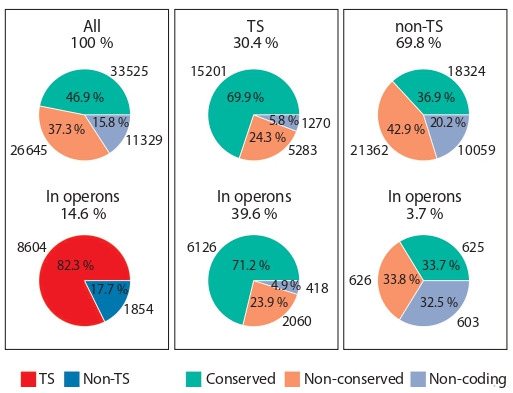
Evolutionary conservation of M. lignano genes. TS – SL trans-spliced; non-TS – non-SL trans-spliced; conserved – proteincoding genes with a homology to human; non-conserved – protein-coding genes lacking the homology to human; non-coding – genes do not code for a protein.

Using these criteria for defining operons, we found that 10 458 genes (approx. 15 % of all genes and 40 % of SL trans-spliced genes) can be assigned to operons, of which 1854 (18 %) start from a non-trans-spliced gene (see Fig. 2, b, Fig. 3). The vast majority of them are comprised of two and three genes (75 and 18 %), with the maximum operon size reaching nine genes (two operons) (see Fig. 2, b). An example of an operon defined in this way is provided in Fig. 2, c.

**SL trans-splicing is enriched in evolutionary conserved and stem cell genes.** We know from a previous study (Grudniewska et al., 2018) that evolutionary conserved proteincoding genes, which still have detectable homology between M. lignano and human, are enriched in somatic stem cells – neoblasts (85 % compared to overall 47 %) (see Fig. 3). On the contrary, only 38 % of germline-specific genes in M. lignano are conserved in human, suggesting their relatively recent appearance in evolution of flatworms. We investigated whether there is a correlation between gene conservation and SL trans-splicing in M. lignano. We found that 69.9 % of the SL trans-spliced genes are conserved between M. lignano and human, while 24.3 % are not conserved and 5.8 % are non-coding (see Fig. 3). Trans-spliced genes that are located in operons have a very similar distribution of conserved, non-conserved and non-coding genes (see Fig. 3). In contrast, among non-trans-spliced genes only 36.9 % are conserved in human, while 42.9 % are non-conserved and 20.2 % are noncoding (see Fig. 3). Thus, SL trans-spliced genes are strongly enriched for conserved genes but there is no dependence on whether these genes are in operons or not.

Next, we calculated the fraction of SL trans-spliced genes among genes enriched in neoblasts (stem cells) and germline – the only proliferation capable cell types in the worm (Grudniewska et al., 2018). Intriguingly, 85 % of the stem cell genes (746) are SL trans-spliced, and almost 86 % (752) are conserved in human (see the Table), and 728 genes are both conserved in human and SL trans-spliced, which is 96.8 % of all the conserved genes in neoblasts. Given that out of 33 525 conserved genes present in the Mlig_3_7 genome annotation 15 201 (45.3 %) are trans-spliced (see Fig. 3), this represents a 2.13-fold enrichment for conserved SL trans-spliced genes among neoblast genes relative to the expected from the random distribution (p-value: 1.98e–7, chi-square test). On the contrary, only 18 % of the germline genes are SL trans-spliced and 37 % are conserved in human. Taken all together, this suggests that SL trans-splicing plays an important role in stem cell regulation in M. lignano.

**Table 1.**
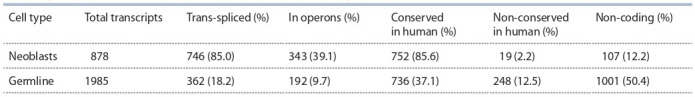
Summary of transcripts from M. lignano proliferation-capable cell types

## Discussion

SL trans-splicing is widespread in diverse flatworm taxa, including both parasitic and free-living species (Zayas et al., 2005; Protasio et al., 2012; Wudarski et al., 2017; Ershov et al., 2019). However, most of the studies of SL trans-splicing were focused on nematodes and trypanosomes (Lasda, Blumenthal, 2011; Lei et al., 2016). Here, we performed the first study which focuses on SL trans-splicing in the free-living regenerative flatworm model M. lignano. By reanalyzing the available genome and transcriptome data, we found that 30 % of the worm genes are SL trans-spliced, and 15 % are estimated to be organized in operons (see Fig. 3). For a comparison, in C. elegans 70 % of genes are SL trans-spliced and 17 % are in operons, in ascidian chordate Ciona intestinalis it is 58 and 20 %, respectively, and in the parasitic liver fluke Schistosoma mansoni 11 % are SL trans-spliced with a few genes in operons (Blumenthal, Gleason, 2003; Satou et al., 2008; Matsumoto et al., 2010; Protasio et al., 2012). Among free-living flatworms, trans-splicing was studied before (Zayas et al., 2005; Rossi et al., 2014), but there is no firm estimation of its abundance and prevalence of genes in operons. The size of operons in M. lignano also varies similarly to C. elegans, where it ranges from two to eight genes, with the most frequent intergenic distance around 100 bp, and the majority of operons comprised of two genes (see Fig. 2) (Allen et al., 2011). 

The most striking finding of our study is that most of M. lignano SL trans-spliced genes are evolutionary conserved (see Fig. 3) and, most importantly, that overwhelming majority of neoblast-specific genes (85 %) are SL trans-spliced (see the Table). Interestingly, 39 % of neoblast genes are also clustered in operons (see the Table), suggesting their early evolutionary origin and the necessity for synchronized expression and similar transcriptional regulation. Neoblasts are the key players of outstanding regeneration capacity in free-living flatworms, and thus they are the primary subject of the studies on flatworm regeneration. All the tissue renewal and growth in adult flatworms is due to neoblast proliferation and differentiation (Egger et al., 2006; Ladurner et al., 2008; Wagner et al., 2011). Our data clearly indicates importance of SL trans-splicing for the gene regulation of neoblasts in M. lignano and lay ground for further studies of how exactly SL trans-splicing machinery contributes to different stages of neoblast activity

## Conclusion

Spliced leader trans-splicing affects a substantial fraction of M. lignano genes. We annotated and characterized the sequence of SL RNA, identified the conserved motifs at the exon-intron/exon-outron junction sites in cis- and SL transspliced genes, and provided the first comprehensive analysis of genes comprising operons in M. lignano. Most importantly, we found that the SL trans-spliced fraction is over-represented by evolutionary conserved protein-coding genes, in contrast to the non-trans-spliced part of the genome, and that the stem cell-specific genes are predominantly SL trans-spliced. Our findings suggest an important and evolutionary conserved role of SL trans-splicing in regulation and maintenance of neoblasts in M. lignano. Thus, a thorough investigation of the molecular mechanism of SL trans-splicing is required to fully understand the regulation of regeneration and stem cell differentiation in flatworms.

## Conflict of interest

The authors declare no conflict of interest.
